# Comparative analysis of immunological biomarkers in COVID-19 and bacterial pneumonia

**DOI:** 10.25122/jml-2023-0273

**Published:** 2023-12

**Authors:** Mudathir Abdelshafea Abdelkareem Abakar, Daralnaeem Hassan Ali Hamad, Eman Faisal, Hashim Mohamed Fad-Alla Omer, Mahmoud Taha Mohamed Faki, Abdellla Esmail Mohammed Idris, Rouwida Omer, Zeinab Osman, Entesar Ahmed Gaffar Elhassan, Mohamed Ahmed Abrahim-Holie, Mohammed Ageeli Hakami, Abdullah Alghamdi, Abdulaziz Alfahed, Ghfren Suliman Aloraini, Nahed Sail Alharthi, Hisham Ali Waggiallah

**Affiliations:** 1 Department of Medical Microbiology and Immunology, Faculty of Medical Laboratory Sciences, Alzaiem Alazhari University, Khartoum, Khartoum State, Sudan; 2 Ministry of Health, Kassala, Kassala State, Sudan; 3 Department of Microbiology, Faculty of Medical Laboratory, University of Kassala, Kassala, Kassala State, Sudan; 4 Medical Laboratory, Diagnostic Center, Kassala, Kassala State, Sudan; 5 Public Health Laboratory, Ministry of Health, Kassala, Kassala State, Sudan; 6 Medical Laboratory, Khartoum, Khartoum State, Sudan; 7 Department of Clinical Laboratory Sciences, College of Applied Medical Sciences Al-Quwayiyah, Shaqra University, Riyadh, Saudi Arabia; 8 Deparment of Medical Laboratory, College of Applied Medical Sciences, Prince Sattam Bin Abdulaziz University, Alkharj, Saudi Arabia

**Keywords:** CRP, C3, C4, IL-8, IL-10, IL-12, TNF-α, IFN-γ, COVID-19, bacterial pneumonia

## Abstract

Coronavirus disease 2019 (COVID-19) is a severe and infectious respiratory condition caused by the Severe Acute Respiratory Syndrome Coronavirus 2 (SARS-CoV-2). This case-control study aimed to evaluate serum levels of various immunological markers in patients with COVID-19 compared to those with bacterial pneumonia and a healthy control group. Serum samples were collected from adult participants across various COVID-19 isolation centers, including Kassala State and Ahmed Gasim Hospital, between April and June 2021. The study included 70 patients diagnosed with COVID-19, 30 with bacterial pneumonia, and 50 healthy controls. Serum levels of C-reactive protein (CRP), complement components C3 and C4, and cytokines IL-8, IL-10, IL-12, TNF-α, and IFN-γ were measured using standard reagent kits. Serum level of CRP was significantly elevated in both bacterial pneumonia and COVID-19 but significantly higher among patients with bacterial pneumonia. C3 and C4 were also increased in both patient groups, with C3 significantly higher in bacterial pneumonia. IL-8, IL-10, IL-12, TNF-α, and IFN-γ were significantly increased in bacterial pneumonia and SARS-Cov-2 compared to healthy controls. However, IFN-γ was significantly increased among patients with COVID-19 than patients with bacterial pneumonia. This study highlights the potential significant impact of COVID-19 on the immunological biomarkers investigated.

## INTRODUCTION

The inflammatory response against Severe Acute Respiratory Syndrome Coronavirus 2 (SARS-CoV-2) is characterized by the production of large amounts of cytokines known as 'cytokine storm', which is closely associated with severe disease, lung damage, multiorgan failure, and mortality [1, 2]. However, cytokine levels vary among individuals with COVID-19, distinguishing between moderate, severe, and recovered cases. Interleukin-6 (IL-6) levels significantly increase in patients with COVID-19, while IL-8 remains relatively low [3]. Furthermore, a range of cytokines and biomarkers, including granulocyte monocyte-colony stimulating factor (GM-CSF), monocyte chemoattractant protein-1 (MCP-1), macrophage inflammatory protein-1alpha (MIP1a), tumor necrosis factor-alpha (TNF-α), interleukin-1 receptor antagonist (IL-1RA), IL-15, and IL-10, have been associated with fatal outcomes in patients with COVID-19 [4]. The dysregulations in antiviral immunity and the elevation of cytokine levels play a critical role in determining the severity of COVID-19 [5].

C-reactive protein (CRP), a marker of inflammation, is significantly elevated in various respiratory virus infections, such as H7N9 and H1N1 influenza, and correlates with the severity observed in COVID-19, as well as in diseases caused by other coronaviruses like Middle East Respiratory Syndrome Coronavirus (MERS-CoV) and the original SARS-CoV [6]. Elevated CRP levels have been linked to increased risk of organ failure and mortality in COVID-19 patients [7, 8].

The role of innate immunity is further underscored by the behavior of mannose-binding lectin, which can bind directly to certain viruses, including SARS-CoV [9]. The serum level of complement proteins differs based on the severity and clinical manifestation of individuals infected with SARS-Cov-2. For instance, complement proteins C3 and C4 increase in non-severe COVID-19 infections and decrease in severe infections [10]. Furthermore, systemic complement activation has been associated with respiratory failure [11].

The types and levels of cytokines also vary depending on the bacterial agent causing pneumonia. TNF is elevated across all causative agents. Furthermore, *Streptococcus pneumonia* and *Legionella* species lead to high levels of IL-6, whereas *Haemophilus influenzae, Moraxella catarrhalis*, and *Chlamydia pneumonia* result in lower cytokine expressions. Elevated interferon-gamma (IFN-γ) is associated with viral and intracellular infections [12]. *Streptococcus pneumoniae* bacteremia is characterized by significantly higher serum IL-8 levels compared to pneumonia caused by *Legionella* species or *Chlamydia pneumoniae* [13]. This study aimed to evaluate the levels of immunological biomarkers such as CRP, C3, C4, IL-8, IL-10, IL-12, TNF-α, and IFN-γ in patients with COVID-19 and bacterial pneumonia.

## MATERIAL AND METHODS

### Study design and participants

This case-control study was conducted between April and June 2021 in several COVID-19 isolation centers, including Kassala State for patients with COVID-19, Ahmed Gasim Hospital, and Khartoum State for patients with pneumonia. This study included 150 adult participants (100 patients and 50 controls), of which 70 (35%) were with patients with COVID-19, 30 (15%) were patients hospitalized with bacterial pneumonia and negative COVID-19 results, and 50 (33.3%) participants were healthy individuals who tested negative for SARS-Cov-2 by PCR. Individuals with lung conditions such as lung nodules, non-small cell lung cancer, small cell lung cancer, and mesothelioma were excluded from the study.

### Methodology

Blood samples were collected from all participants. Serum concentrations of CRP, C3, and C4 were measured in mg/dl using Aptec Diagnostics reagent kits. These measurements were carried out using the Biosystem A15 automated chemical analyzer, a clinical laboratory tool designed to rapidly assess various chemicals and biological samples with minimal human intervention, which can aid in disease diagnosis. Cytokine levels (IL-8, IL-10, IL-12, TNF-α, and IFN-γ) in pg/ml were quantified using ELISA with Abcam^®^ ELISA reagent kits.

Data analysis was conducted using SPSS software. Descriptive statistics were calculated to summarize the dataset, including means, medians, standard deviations, frequencies, and percentages. We used the Kruskal–Wallis test to compare median biomarker levels and the Mann–Whitney U test for pairwise comparisons when significant differences were detected. The Chi-square test was used for categorical data analysis. The reference values for the biomarkers used in this study, based on the standard ranges of the kits, were CRP up to 10 mg/dl, complement components C3 and C4 at 75 - 135 mg/dl, and 9 - 36 mg/dl, respectively. For cytokines, IL-8 was set at 3.1 - 13.6 pg/ml, IL-10 at 0 - 2.8 pg/ml, IL-12 below 3.0 pg/ml, TNF-α at 0 - 2.8 pg/ml, and IFN-γ at 0 - 4.2 pg/ml.

## RESULTS

This study included 150 participants, of whom 70 (46.7%) were diagnosed with COVID-19 during their first five days of illness, 30 (20%) were hospitalized for bacterial pneumonia, and the remaining 50 (33.3%) were healthy controls. The COVID-19 group included only male participants. Participants ranged in age from 18 to 90 years old, with a mean age of 42.32 years.

### Serum complement levels

Table 1 shows a significant increase in serum C3 and C4 levels in patients with COVID-19 (P<0.000) and pneumonia (P<0.000) as compared to the healthy control group. Despite the slight variation in C4 levels, the increase in C3 levels among the bacterial pneumonia group was comparable to that observed in the COVID-19 group (P<0.001)

**Table 1:** CRP, C3, and C4 across COVID-19, bacterial pneumonia, and control groups

Marker		(A) COVID-19 (n=70)	(B) Bacterial pneumonia (n=30)	^1^(C) Control (n=50)	P value*	Multiple P values**
CRP	Median	7.6	31	2.25	0.000	AXB 0.000 AXC 0.000 BXC 0.000
	Mean	20.07	45.73	3.26		
	SD	27.76	40.72	2.77		
	Min	.3	.5	.4		
	Max	125.0	140.0	10.1		
C3 mg/dl	Median	100.30	129.30	19.75	0.000	AXB 0.001 AXC 0.000 BXC 0.000
	Mean	102.41	123.55	21.18		
	SD	24.55	50.98	7.26		
	Min	52.20	11.40	12.30		
	Max	167.10	201.50	45.50		
C4 mg/dl	Median	23.25	25.60	14.20	0.000	AXB 0.383 AXC 0.000 BXC 0.000
	Mean	23.92	24.65	14.25		
	SD	10.40	7.19	2.64		
	Min	7.20	11.40	8.90		
	Max	56.50	37.50	21.0		

* Kruskal–Wallis test was used to calculate the P value; **Mann–Whitney U test was used to calculate the P value; A P value less than 0.05 is considered significant; ^1^ control for C3 and C4 (n=50); A, B, and C are vectors of the scalar triple product; SD standard deviation; n number

### C-reactive protein

This study found a significant increase in the median serum level of CRP across all groups (P<0.000), with significantly higher values in individuals with bacterial pneumonia compared to COVID-19 (P<0.000) and the control group (P<0.000). Compared to healthy controls, the median CRP of patients with COVID-19 was considerably higher (P<0.000) (Table 1). Specifically, 86.7% of patients with bacterial pneumonia and 62.9% with COVID-19 had elevated levels of CRP compared to 24% of healthy controls (Table 2). Furthermore, CRP concentrations above 80 mg/dl and 100 mg/dl were significantly more prevalent in the bacterial pneumonia group compared to the moderate COVID-19 cases.

**Table 2:** The relationship between CRP and severity of COVID-19 and bacterial pneumonia

CRP	(A) Mild COVID-19 (n=22)	(B) Moderate COVID-19 (n=48)	(C) Bacterial pneumonia (n=30)	(D) Control (n=50)	P value	Multiple P values
>10 mg/dl	12(54.5%)	32(66.7%)	26(36.7%)	24(24%)	0.035	AXB 0.330 AXC 0.010 AXD 0.004 BXC 0.049 BXD 0.000
>20 mg/dl	7(31.8%)	17(35.4%)	24(80%)	0(0.00%)	0.000	AXB 0.768 AXC 0.000 AXD 0.000 BXC 0.000 BXD 0.000
>40 mg/dl	3(13.6%)	6(12.5%)	10(33.3%)	0(0.00%)	0.057	AXB 0.895 AXC 0.105 AXD 0.000 BXC 0.027 BXD 0.000
>80 mg/dl	0(0.00%)	5(10.4%)	7(23.3%)	0(0.00%)	0.034	AXB 0.116 AXC 0.015 BXC 0.124 AXD ----- BXD 0.001
>100 mg/dl	0(0.00%)	3(6.3%)	6(20%)	0(0.00%)	0.029	AXB 0.231 AXC 0.026 BXC 0.064 AXD ----- BXD 0.012

The chi-square test was used to calculate the P value/ P value less than 0.05 considered; A, B, and C are vectors of the scalar triple product; n: number

**Figure 1:**
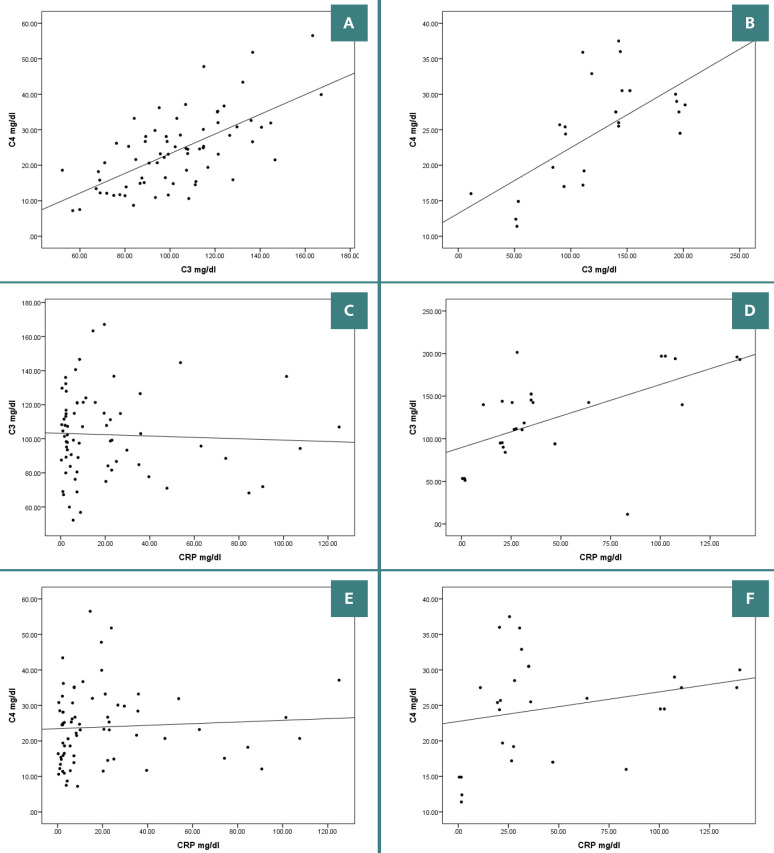
The association between CRP, C3 and C4. A: Correlation between C3 and C4 in the COVID-19 group (R=0.654; P<0.000); B: Correlation between C3 and C4 in the bacterial pneumonia group (R=0.657; P<0.000); C: Correlation between CRP and C3 in the COVID-19 group (R=-0.045; P<0.710); D: Correlation between CRP and C3 in the bacterial pneumonia group (R=0.591; P<0.001); E: Correlation between CRP and C4 in the COVID-19 group (R=-0.063; P<0.607); F: Correlation between CRP and C4 in the bacterial pneumonia group (R=0.236; P<0.210).

Table 3 reveals that normal levels of C3 were found significantly more often in patients with COVID-19 (75.7%) compared to those with pneumonia (33.3%), indicating a potential relationship between normal C3 levels and COVID-19. In contrast, patients with pneumonia had a greater incidence of C3 abnormalities, either elevated or reduced. Abnormal C4 levels were more prevalent among patients with COVID-19. However, this finding did not reach statistical significance (P=0.101). Further analysis indicated that most patients with bacterial pneumonia (46.7%) had elevated C3 levels, with C4 levels remaining within the normal range. Conversely, patients with COVID-19 had extreme outcomes of low C3 and C4 and high C3 and C4. The comparison group had normal C3 and C4 levels (Table 4). There were significant relationships in C3 and C4 levels between COVID-19 and pneumonia groups (Figure 1 A-B).

**Table 3:** Association between C3 and C4 and study groups

Complement	COVID-19 (n=70)		Pneumonia (n=30)		P value	
	Normal	Abnormal	Normal	Abnormal		
**C3**	**53(75.7)**	**17(24.3)**	**10(33.3)**	**20(66.7)**	**0.000**	
**C4**	**59(84.3)**	**11(15.7)**	**29(96.7)**	**1(3.3)**	**0.101**	
		**C3**			**C4**	
**Study groups**	**Low**	**Normal**	**High**	**Low**	**Normal**	**High**
**COVID-19 (n=70)**	**9 (12.9)**	**53 (75.7)**	**8 (11.4)**	**3 (4.3)**	**59 (84.3)**	**8 (11.4)**
**Pneumonia (n=30)**	**5 (16.7)**	**10 (33.3)**	**15 (50.0)**	**0 (0.0)**	**29 (96.7)**	**1 (3.3)**

**Table 4:** Distribution of C3 and C4 across study groups

Study groups	C3 and C4						
	N C3 N C4	N C3 L C4	N C3 HC4	L C3 N C4	L C3 L C4	H C3 N C4	H C3 H C4
COVID-19 (n=70)	47(67.1)	1(1.4)	5(7.1)	7(10.0)	2(2.9)	5(7.1)	3(4.3)
Pneumonia (n=30)	10(33.3)	0(0.0)	0(0.0)	5(16.7)	0(0.0)	14(46.7)	1(3.3)

N=Normal; L=Low; H=High

There was a strong association between C3 and CRP in patients with bacterial pneumonia (R=0.591; P<0.001) and a weak negative correlation with COVID-19 (Figure 1 C-D). CRP and C4 had an insignificant positive association with bacterial pneumonia and an insignificant weak negative correlation with COVID-19 (Figure 1 E-F).

### Cytokine levels

The highest serum levels of IL-8 were detected in patients with bacterial pneumonia, with no significant increase in COVID-19 (P=0.592). Significant differences were observed within groups (P<0.000), with notably lower levels in the healthy control group compared to both groups (P<0.000) (Table 5). Regarding IL-10, patients with COVID-19 had the highest serum levels, followed by bacterial pneumonia, and the lowest levels were seen in healthy controls. These variations among the groups were statistically significant (P<0.000). Although there were significant differences between healthy controls, COVID-19 (P<0.000), and bacterial pneumonia (P<0.000) groups, there was no significant difference between patients with COVID-19 and bacterial pneumonia (P=0.755). A significant intergroup P value (P<0.000) was observed when comparing IL-12 levels between study groups. The median differences between IL-12 levels in COVID-19 and bacterial pneumonia were small (P=0.379). However, there were significant differences between these patient groups and healthy controls (P<0.000).

**Table 5:** Comparative analysis of serum cytokine levels among COVID-19, bacterial pneumonia, and healthy control groups

Cytokines	(A) COVID-19 (n=70)	(B) Bacterial pneumonia (n=50)	(C) Control (n=50)	P value *	Multiple P values **
IL-8 pg/ml	143.23	204.80	19.50	0.000	AXB 0.592 AXC 0.000 BXC 0.000
IL-10 pg/ml	21.57	16.57	11.22	0.000	AXB 0.755 AXC 0.000 BXC 0.011
IL-12 pg/ml	663.80	491.52	51.88	0.000	AXB 0.379 AXC 0.000 BXC 0.000
TNF-α pg/ml	230.81	246.22	75.09	0.000	AXB 0.316 AXC 0.000 BXC 0.000
IFN-γ pg/ml	59.58	27.40	14.45	0.000	AXB 0.001 AXC 0.000 BXC 0.006

* Kruskal–Wallis test was used to calculate the P value; ** Mann–Whitney U test was used to calculate the P value; A P value less than 0.05 is considered significant; A, B, and C are vectors of the scalar triple product; n: number

TNF-α levels were increased in both bacterial pneumonia and COVID-19 groups, although this difference was not statistically significant (P=0.316). However, TNF-α levels were significantly higher in COVID-19 and bacterial pneumonia groups compared to healthy controls (P<0.000) (Table 5). Serum IFN-γ levels were significantly higher in patients with COVID-19 compared to both bacterial pneumonia groups (P=0.001) and healthy controls (P<0.001). The difference in IFN-γ levels between bacterial pneumonia and healthy controls was also significant (P=0.006).

## DISCUSSION

The present study illustrated significant increases in median CRP levels in patients with bacterial pneumonia compared to patients with COVID-19 and the control group. As far as we know, there is no published data comparing CRP levels between bacterial pneumonia and COVID-19, but generally, CRP is more elevated with bacterial than viral infections [14, 15]. Bhuiyan *et al*. [16] reported a significant increase in the median CRP of patients with bacterial pneumonia compared to patients with viral pneumonia and concluded the importance of combining clinical manifestations with biomarkers rather than using CRP alone to differentiate bacterial from viral pneumonia. Higdon *et al*. [17] also found a significant positive association between bacterial pneumonia and increased CRP, contrary to respiratory syncytial virus (RSV), which showed a negative association. In this study, the median CRP mg/dl of patients with COVID-19 was significantly increased compared to the healthy control, with a greater increase among patients with moderate clinical manifestations than those with mild disease. Elevated levels of CRP have been observed in patients with COVID-19 and are recognized as an early biomarker for predicting disease severity [18, 19]. The variation in CRP concentration can be attributed to the severity of the disease [7, 8]. Pink *et al*. reported higher median CRP levels in patients treated in intensive care units (ICUs) than in non-ICU settings [20]. Another study by Fazal *et al*. [7] indicated that severe cases and non-surviving COVID-19 patients had greater increases in CRP levels than their non-severe or surviving counterparts. The present study showed significant increases in serum concentrations of CRP >10, >20, >40, >80, and >100 mg/dl in patients with bacterial pneumonia. CRP >20 could be the cut-off point separating the healthy population from patients with bacterial pneumonia and COVID-19. Moreover, CRP levels above 80 mg/dl could differentiate mild COVID-19 cases from bacterial pneumonia, while levels above 100 mg/dl were significant in distinguishing moderate COVID-19 cases from bacterial pneumonia. Higdon *et al*. [17] considered CRP at 37.1 mg/dl as a discriminatory cut-off point between bacteria and RSV pneumonia, with better specific discrimination at a CRP >100 mg/dl cut-off point.

The present study illustrated a significant increase in C3 and C4 among patients with COVID-19 and bacterial pneumonia compared to healthy controls. This indicates activation of classical, alternative, and/or mannose-binding lectin pathways of the complement system in response to these diseases. Similar results were obtained by Bagherimoghaddam *et al*. [21], who reported higher levels of C3 and C4 in patients with COVID-19 than their normal ranges. Another study by Holter *et al*. [11] mentioned an increase in complement activation above the upper limits of normal ranges during admission time. This study showed that the activity of C3 was stronger in bacterial pneumonia than in COVID-19, whereas the activity of C4 was stronger with COVID-19. Such patterns suggest the involvement of the classical and mannose-binding lectin pathways, potentially mirroring the immune response to the SARS-CoV-2 virus as previously seen with SARS-CoV [22]. Most participants in this study showed normal levels of C3 and C4.

Furthermore, 67.1% of patients with COVID-19 and 33.3% of patients with bacterial pneumonia were admitted with normal levels of C3 and C4. These findings are consistent with those of Shaldoum *et al*. [23], who reported that 57.14% of their total study population had normal complement levels, and 71.42% and 57.14% had normal C3 and C4 levels, respectively. The prevalence of abnormal C3 results over C4 in this study underscores the potential role of C3 as a pivotal factor in COVID-19 pathogenesis. This conclusion is partially supported by the work of Cheng *et al*. [24], which identified increased C3 concentration as a risk factor for severe outcomes in younger COVID-19 patients [24]. Furthermore, this study identified that patients with COVID-19 were more likely to have extreme levels of both low and high C3 and C4. Elevated levels of these proteins could result from activation through multiple pathways, whereas reduced levels might indicate immune deficiency or the depletion of these proteins due to the formation of immune complexes [14, 22]. We observed significant correlations between serum levels of complement components C3 and C4 among patients with COVID-19 and bacterial pneumonia. In the bacterial pneumonia group, there were significant and insignificant positive correlations between CRP and complement proteins C3 and C4, respectively, suggesting a potential role of CRP in activating the complement system during bacterial infection. However, COVID-19 patients had a weak negative correlation that was not statistically significant. This may indicate the important role of CRP as an activator of the complement system in bacterial infection.

IL-8 levels were significantly elevated in bacterial pneumonia and COVID-19 groups compared to controls, aligning with Bohnet *et al*. [25], who identified elevated IL-8 levels in patients with pneumonia [25]. In addition, Kragsbjerg *et al*. [13] found that *Streptococcus pneumoniae* was associated with significant increases in serum IL-8 compared to other bacterial pneumonia. This may refer to the ability of the C3-binding protein of *Streptococcus pneumoniae* to stimulate the production of IL-8 [26], which is involved in neutrophil accumulation and pathogenesis [27].

Similarly, increases in IL-10 were significant among patients with COVID-19 and bacterial pneumonia, with the COVID-19 group showing a higher, though not statistically significant, increase. Community-acquired pneumonia is characterized by an increased level of IL-10 in the serum and lung, and the severity of the disease is associated with systemic IL-10 elevation [28]. The increases in IL-10 in the lung could interfere with neutrophil accumulation and promote secondary bacterial infections such as *Streptococcus pneumoniae* after influenza [29], as well as insufficient clearance of bacteria from the lung [30]. Our data also indicated that IL-12 levels were significantly higher in the COVID-19 than in the bacterial pneumonia group. This regulatory cytokine, produced by macrophages and B-lymphocytes, plays a crucial role in the immune response by promoting the generation of TH1 subset CD4+ lymphocytes and activating natural killer cells [31]. It induces the production of IFN-γ by T lymphocytes and natural killer cells, thereby enhancing the immune response against intracellular bacteria [32]. IL-12 may contribute to the killing of intracellular bacteria by promoting IFN-γ production and extracellular killing through the subsequent production of nitric oxide (NO) by phagocytes [31]. Our findings align with previous studies that reported a significant elevation of IL-12 among patients with COVID-19 compared to control groups [33, 34].

Furthermore, the serum level of TNF-α was elevated in bacterial pneumonia and COVID-19 groups, although not statistically significant. This finding is consistent with the results reported by Wang *et al*., who observed a significant increase in serum TNF-α levels among patients with bacterial pneumonia [35]. Similarly, Qingqing *et al*. reported a significant increase in TNF-α levels among patients diagnosed with COVID-19 compared to individuals without COVID-19 [36]. Finally, the present study showed significantly increased levels of serum IFN-γ in both bacterial pneumonia and COVID-19 groups compared to the healthy control group. This finding aligns with the results reported by Kragsbjerg *et al*. [37], who also observed an increase in IFN-γ levels in patients with viral and bacterial pneumonia. Elevated levels of IFN-γ have been associated with a poor prognosis [38], whereas decreased levels represent a risk factor for lung fibrosis among patients with SARS-CoV-2 infection [38].

## CONCLUSION

Both bacterial pneumonia and COVID-19 significantly influenced all inflammatory mediators used in this study, suggesting their potential use as potent biomarkers for diagnosis. IFN-γ was significantly increased in patients with COVID-19 compared to patients with bacterial pneumonia, indicating its potential as a biomarker for early diagnosis and differentiation from bacterial pneumonia. Further investigation is recommended to include severe or ICU patients and to measure these parameters at different time intervals during the patient's stay in the isolation center.
